# Bronchopneumonia

**Published:** 1904-12

**Authors:** 


### BRONCHOPNEUMONIA.

W. P. Northrup, in the *Medical News* for April 30, thus summarizes *how to cure a baby with bronchopnenmonia:* (1) Castor oil to clear the field of operation. It is the first aid to the injured.

(2) Fresh air, cool and flowing. It reddens the blood, stimulates the heart, improves digestion, quiets restlessness, aids against toxemia. Regulate the temperature of air of the room inversely to that of the child. The patient's feet should always be warm, and the head cool. (3) Water plenty, inside and outside, temperature of the water as indicated by child's temperature. (4) Quiet and rest. Tranquilizing influences about patient. Undisturbed sleep. (5) Correct feedings to avoid fermentation and gas in abdomen. If there is need, high hot salines. (6) Antipyretics. Water, no coal-tar products. (7) Heart stimulants. Fresh air, hot foot-baths. Relieving tympanites and crowding. Hot foot-baths and hot salines can be given in a cold room. Both can be given under the bed clothes. Drugs, whiskey, and strychnin; promote general comfort in every rational way. *How to kill a baby with broncho-pneumonia:* Crib in far corner of the room with canopy over it. Steam kettle; gas stove (leaky tubing); room at 80° F.; many gas jets burning. Friends in the room, also the pug dog. Chest tightly enveloped in waist-coat poultice. If child's temperature is 105° F. make poultice thick, hot and tight. Blanket the windows, shut the doors. If these do not do it, give coal-tar antipyretics and wait.

